# Family happiness among people in a Southeast Asian city: Grounded theory study

**DOI:** 10.1111/nhs.12688

**Published:** 2020-02-19

**Authors:** Wilai Napa, Jumpee Granger, Siranee Kejkornkaew, Pornsiri Phuagsachart

**Affiliations:** ^1^ 270 Ramathibodi School of Nursing, Faculty of Medicine Ramathibodi Hospital Mahidol University Bangkok Thailand

**Keywords:** family, grounded theory, happiness, Thailand, city

## Abstract

Happiness is a fundamental human aspiration. However, many families live in places where the cost of living is high, and there is a need for people to balance their lives to maintain happiness. This study explored the characteristics of happiness for families living in a city in Thailand. A grounded theory methodology was used to collect and analyze data. Thirteen participants were recruited into the study by purposive sampling based on a set of inclusion criteria, followed by theoretical sampling. The findings demonstrated that living together without financial problems was a core category of family happiness, which involved close connections, caring for one another and financial security. Families needed to balance these components when facing stress, using coping methods to restore happiness. To enhance family happiness, health professionals should focus on caring and communication within families, including coping techniques to maintain family happiness.

## 1 INTRODUCTION

Happiness is a fundamental human aspiration. Individual happiness can be defined as a person's expression of positive emotions and the delivery of personal happiness to others using their individual strengths and virtues (Seligman, 2002). Happiness refers to an individual's judgment about satisfaction with their entire life, which depends on the person's levels of satisfaction, self‐perception of goal attainment, and evaluation of overall life impact (Veenhoven, 2015). Previous studies have reported that positive emotions, feelings of gratitude, leisure activity, and social comparisons determine individual happiness (Peterson, Ruch, Beermann, Park, & Seligman, 2007; Richard et al., 2015; Shimai, Otake, Park, Peterson, & Seligman, 2006; Yiwei, 2013). Previous research has indicated that family factors, including family characteristics, circumstances, and distance from the city, can influence individual happiness. One study examined how family characteristics, such as financial prosperity, good relationships, family success, family cohesion, performance of family activities, and maintaining family health, are related to individual happiness (Stasova & Vilka, 2018). Various studies have reported that family circumstances, such as spending time together and living with family members (Gray, Chamratrithirong, Pattaravanich, & Prasartkul, 2013), caring for one another (Kramanon & Gray, 2015), maintaining healthy family relationships (Chiang & Lee, 2018; Nanthamongkolchai, Tuntichaivanit, Muangsawaengsub, & Chrupoonphol, 2009) and family satisfaction (Sesanu & Singhapakdi, 2014), influence individual happiness. A range of studies have also reported that the happiness of rural residents is generally higher than that of urban or city residents (Helliwell, Shiplett, & Barrington‐Leigh, 2018; Nozhnitskiy & Naples, 2014). Moreover, the cost of living is related to the happiness of people living in the city (Knight & Gunatilaka, 2018). These findings suggest that living closer to nature can result in lower levels of stress for people living in rural areas. The family factors reported by previous studies to affect on individual happiness mentioned here have typically been measured using questionnaires. However, little is currently known about perspectives of family happiness expressed through in‐depth interviews.

The United Nations surveyed happiness across countries and constructed rankings using the Happiness Index, reporting that Thailand ranked 36th among 156 countries (Helliwell & Wang, 2013). Previous studies demonstrated that many factors, including income, debt, homeownership, education level, physical health, perceived neighborhood quality, and sense of income satisfaction, could predict happiness among people in Thailand (Gray, Kramanon, & Thapsuwan, 2008). One study reported that income was important for Thai people with low economic status in terms of happiness and living in the context of economic inequality (Royo, Velazco, & Camfield, 2007). Thus, people in Thailand with a high cost of daily living appeared to be less happy than others. Another study reported that family satisfaction and community satisfaction were significantly associated with Thai workforce happiness (Tangsathapornphanich, Senasu, & Sakworawich, 2017). Moreover, a survey of 200 Thai volunteers in the central region reported a happiness level of 69.1% and found that familial happiness was a primary dimension of individual happiness (Soopunpitug, 2017). This research appears to indicate that family happiness influences individual happiness among people in Thailand. Thus, happiness among people in Thailand has been examined using various dimensions, particularly factors influencing on individual happiness. However, studies investigating the characteristics of family happiness in Thailand remain scarce. Therefore, it may be valuable to study Thai perspectives of the characteristics of family happiness, particularly among those who have migrated to the capital city from other regions (Helliwell, Shiplett, & Barrington‐Leigh, 2018; Nozhnitskiy & Naples, 2014).

### 1.1 International perspectives

Individual happiness can be represented by various characteristics. Happy people tend to express positive emotional expressions, to have a sense of gratitude, and to have a tendency to spread happiness to others (Seligman, Parks, & Steen, 2004). Happy people gain pleasure in life by learning from their experiences and living meaningful lives. Various studies have demonstrated that individual personality factors influence happiness (Peterson et al., 2007; Richard et al., 2015). One previous study found that positive emotions and a sense of gratitude were associated with happiness among both American and Japanese young adults (Shimai et al., 2006).

Furthermore, gratitude among American adults has been reported as a robust predictor of life satisfaction, whereas perseverance was reported to be the most frequently encountered predictor of life satisfaction among people in Switzerland (Peterson et al., 2007). This finding suggests that individual personality characteristics can influence happiness, and that these effects may vary in different contexts. Self‐assessment of life and social comparisons with other adults are reported to be the strongest predictors of happiness among Chinese adults (Yiwei, 2013). In a survey comparing computer literacy among older persons, happiness was found to be quickly gained through simple activities in daily life, unlike happiness among younger people (Bhattacharjee & Mogillner, 2014). Happiness is also likely to have an association with physical leisure activities (Richard et al., 2015). Overall, it has been reported that happiness varies with age, activities, and social comparisons.

Other factors, such as house location, employment, family circumstances, and familial happiness, are also known to affect individual happiness. Previous studies reported that house location, employment status (Mehrdadi, Sadeghian, Direkvand‐Moghadam, & Hashemian, 2016), marital status, educational level, income, and informal and formal social participation were all related to happiness (Azizi, Mohamadian, Ghajarieah, & Direkvand‐Moghadam, 2017). Income also contributes to urban dwellers' happiness, whereas distance from the city and family members were found to predict rural dwellers' happiness in Greece (Liltsia, Michailidisa, & Partalidoua, 2014). Findings from China have consistently indicated that rural–urban migrant happiness is influenced by household income and expected income over the next 5 years (Knight & Gunatilaka, 2018). This finding may suggest that economic factors matter more in the happiness of urban people than rural dwellers. Several studies have reported that Americans and Canadians living in large cities are likely to be less happy than those living in mid‐sized or small centers (Helliwell, Shiplett, & Barrington‐Leigh, 2018; Nozhnitskiy & Naples, 2014). In addition, well‐being, quality of life, and a sense of community have been found to be significant contributors to happiness in community living (Helliwell, Shiplett, & Barrington‐Leigh, 2018). Previous studies in Thailand have demonstrated that familial happiness is a primary dimension of individual happiness (Soopunpitug, 2017) as well as family satisfaction (Tangsathapornphanich et al., 2017).

Overall, the previous studies mentioned indicate that personality characteristics and other factors, such as family circumstances, socioeconomic, social participation, distance from the city, and family happiness, influence individual happiness. However, little is currently known about city‐dwellers' perceptions of family happiness. Understanding perceptions of family happiness can enable health professionals to provide adequate family care and other resources to enhance happiness among families living in the city, potentially increasing individuals' happiness.

## 2 RESEARCH OBJECTIVES

This study aimed to investigate healthy Thai individuals' beliefs about the characteristics of family happiness in the city.

## 3 METHODS

### 3.1 Study setting

The setting for this study was a community located in Bangkok, the capital city of Thailand, consisting of both residents and immigrants who had moved from other provinces. The research was conducted at the residents' homes or in places selected as comfortable by the participants, such as homes or workplaces. The data were collected from December 2016 to August 2017.

### 3.2 Research design

This study used the grounded theory method of Strauss and Corbin (1998) to explore Thai perspectives relating to the characteristics of family happiness. This methodology has its roots in symbolic interactionism, which focuses on the process of interpersonal relationships through social interpretation. We conducted interviews in which Thai people living with their families were able to express their beliefs about the characteristics of family happiness, which are interpreted through social interactions and influenced by historical and cultural contexts. Family happiness is a dynamic process subject to change based on variations in context. Therefore, the grounded theory methodology provided a suitable approach for gaining understanding about family happiness. We conducted open coding, axial coding, and selective coding while analyzing data, and constant comparative analysis was used to identify similarities or differences across the data. This study applied theoretical sampling for participant selection during data collection, to obtain depth and richness of data (Saunders et al., 2018). These methods enabled the concepts emerging from participants' perspectives to be more precise, concise, and relevant to family happiness.

### 3.3 Ethical considerations

In compliance with the World Medical Association Declaration of Helsinki, this study was approved by the Institutional Review Board of Ramathibodi Faculty of Medicine (ID: 08‐59‐61) for conducting research involving human subjects. Before interviewing the participants, the researchers sought permission by providing information regarding the benefits, confidentiality, possible hazards, and freedom to withdraw from the project to all of the participants. Participants then permitted the researchers to conduct interviews with audio recording by signing informed consent forms.

### 3.4 Participants and recruitment

All participants were aged 18 years or older, had lived with family members for up to 1 year, spoke Thai, and did not have severe physical and/or psychological problems. The researchers selected participants using purposive sampling based on set inclusion criteria and eligibility. The recruitment procedure initially accessed participants who met the inclusion criteria by referral from primary care unit staff. The primary care unit was located in the community and participants were able to access it at times that suited them or when they needed health care. The researchers also used theoretical sampling to select additional participants based on the concepts obtained from the interviews to guide subsequent participant recruitment (Saunders et al., 2018). For example, the initial participants explained that they had moved from other provinces to Bangkok for work and were familiar with the people around them in the neighborhood, which made them happier. Based on their descriptions, the researchers subsequently recruited participants who were original residents, to compare their happiness with that of residents who had recently migrated. Unhappiness in families was also used to inform theoretical sampling to recruit another participant to test the formulation of concept grounding from data (Bowen, 2008). For example, the researchers selected a family member who was a housekeeper and took care of an ex‐husband with paralysis and an older unemployed son. Her description presented the emotional components of unhappiness in the family, diverging from the emerging theme of family happiness. Therefore, this method helped the researchers obtain a more in‐depth and broader concept of family happiness.

### 3.5 Data collection and analysis procedures

This study used in‐depth interviews to explore individual perspectives about the characteristics of family happiness. Semistructured interview questions were used to explore participants' views, such as “What do you feel about your family?” “What are your thoughts about family happiness?” “What does a happy family look like?” “How do other family members help you when you are faced with trouble?” and “What or how do you gain and maintain family happiness?” These questions were developed based on a review of previous research on happiness. However, the researchers modified the questions to explore perspectives of family happiness. The researchers used probing questions for greater exploration of the participants' views to gain valuable information about family happiness. Probing questions helped the researchers understand family happiness and obtain redundant data; consequently, the data reached saturation (Legard, Keegan, & Ward, 2003). Each participant was interviewed for approximately 45–60 min, and audio recordings of the responses were collected. In addition, the researchers maintained sensitivity regarding participants' nonverbal cues and verbal communication while conducting the interviews.

Each interview was transcribed verbatim by a transcriber, and the researchers corrected the data from the transcripts and audio recordings to ensure that the transcripts correctly recorded local dialects. After completing the transcripts, the researchers read and re‐read the transcripts of the interviews until they felt that they understood what the participants had said. Open coding was initially started line‐by‐line, and the descriptions were coded using vivo coding (actual spoken words or phrase of participant). Next, the researchers read the coded data and grouped the content using the axial coding process. In this process, the researchers used a conditional matrix to verify the relationships among the groups (Table 1). In addition, the researchers went back and forth between open coding and axial coding several times until the emerging category was condensed in terms of properties and dimensions. Finally, the groups of axial coding were analyzed by determining how they explained correlations among all coding in the sense of family happiness, and selected coding emerged from all of the coding. When faced with discrepancies in coding during the analysis, the researchers held discussions until they reached agreement. Furthermore, the researchers systematically applied the constant‐comparative method to obtain condensed categories covering all family happiness dimensions at every stage of data collection and analysis.

**Table 1:** Example of conditional matrix table

Participants' description	Open coding		Axial coding
	Vivo code	Conceptual coding	
We regularly have dinner together at evening. We also spend time on a weekend to cook and eat together.	Regularly cook and eat together	Spending time together	Close connection
We cook and eat together on weekend. Our relatives living around also gather here to eat and talk to another.	Eat together	Spending time together	Close connection
After I pay for food, miscellaneous expenses, I'll put the rest of the money to the piggy bank every day. This leads me to have a big amount of money in future.	Putting money in a piggy bank	Saving money	Financial security
We have to carefully think about our expense. (We) buy things a reasonably as possible as we can do.	Buying things at a reasonable cost	Thinking ahead for expenses	Financial security

### 3.6 Trustworthiness

To maximize the credibility of the data, the researchers frequently discussed emerging categories and subcategories, achieving agreement regarding the data analysis through peer debriefing. Member checking was also used to increase the accuracy of data (Kolb, 2012). Researchers then presented all categories and subcategories to the participants to determine whether they had shared all of their experiences regarding family happiness and to confirm the emerging categories. These procedures ensured that all categories and subcategories had emerged from the raw data based on the participants' views. The study included participants with diverse backgrounds, to gain broader perspectives of family happiness (Bowen, 2008). These procedures enhanced the emerging categories, met transferability requirements, and may be useful for explaining the family happiness of people living in a city.

## 4 RESULTS

Overall, 13 participants were recruited for the research project. All participants were low‐income earners, with the following occupations: food vendors (5), tailors (2), housekeepers (2), a dental assistant (1), a motorcycle taxi driver (1), a security guard (1), and a grocery store owner (1). Participants lived in nuclear and extended families.

### 4.1 Core category: Living together without financial problems

To formulate the characteristics of family happiness emerging from the descriptions, the core category “living together without financial problems” was found to reflect family happiness, particularly among city‐dwelling people in Bangkok. Family happiness was defined as family satisfaction that family members lived together and took care of one another as a familial responsibility. Additionally, happy families were those that did not face financial constraints. Family happiness consisted of three categories of features: close connections, caring for one another, and financial security. Family happiness increased or decreased when family members were faced with stress.

**Figure 1:**
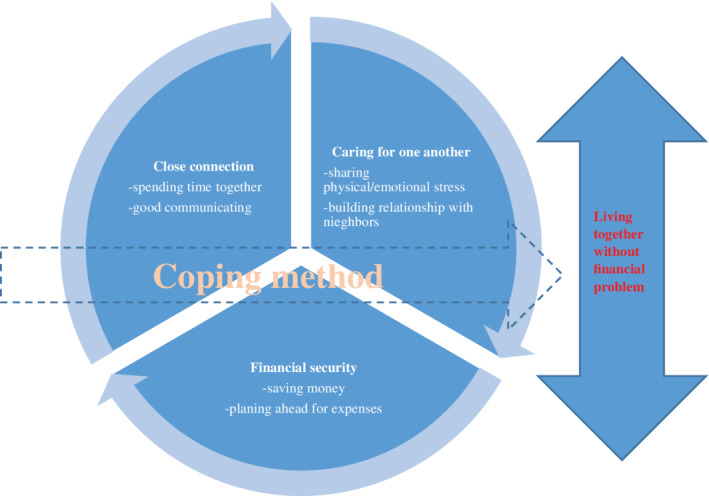
The family happiness process among Thai family

Figure 1 shows that the three categories, including close connection, caring for one another, and financial security, operated within contextual conditions, and each category involved actions taken by families for pursuing their happiness. Consequently, families were able to achieve happiness and live together without financial difficulty. However, if the family faced difficulty in daily life, this impacted contextual factors. The family applied coping methods to resist stress and restore their happiness. This process of family happiness shifted over time. Each component is described in more detail next:

**Category I:** Close connections can be defined as situations in which family members engage in activities and spend time together by having dinner, taking short trips together, or visiting other relatives. This category represents the closeness of family members to each other, possibly indicating that families achieve cohesion through these activities. Family members with close connections also tended to have friendly discussions and avoided arguments.

*Subcategory I, spending time together*. Families regularly spent time together having dinner, taking short trips, or visiting other relatives. For example:

“My family has three members. We frequently travel together during traditional festivals such as New Year and Songkran (Thai New Year). We also go to visit other relatives who live far away.” (Security guard, nuclear family)

*Subcategory II, good communicating*. Participants expressed that they did not use harsh words or argue with one another. They felt that these positive conversations made them happier. For example:

“We don't have any arguments…we talk to each other in a friendly manner and dine together. This is happiness…this makes me happy” (Food vender 1, extended family)

In this category, the results suggested that happy families have close connections with one another, in which they spend time together with positive communication among family members. Consequently, these behaviors can lead to a pleasant family atmosphere and increased family happiness.

**Category II:** Caring for one another can be defined as the ways in which family members shared their concern for one another by relieving physical and emotional strain. In addition, the participants' descriptions revealed how family members built relationships outside the family by giving gifts and watching out for one another's safety.

*Subcategory I, sharing physical and emotional stress*. Participants reported that other family members understood them when they experienced stress. Other family members were alongside them when they needed them to be and provided encouragement. For example:

“(My older brother and sister) Care for me…they don't leave me by myself. So, I have to take care of my father instead of them. Anyway, they care for me, too. We care for one another.” (Housekeeper, nuclear family)

*Subcategory II, building relationships with neighbors*. Another behavior associated with increased family happiness was building good relationships with neighbors. Some neighbors kept watch for participants' families' safety when they were away from home for work. These practices helped families develop good relationships outside the family. For example:

“My neighbors always keep watch over my elderly parents and house. They do that when I leave my parents alone or go out to work. If they hear a noise from my house, they will visit my parents and find out how they can help.” (Dental assistant, nuclear family)

These comments reflect how family members cared for one another, sharing physical and emotional strain by giving one another support. As a result, they derived happiness from other family members' encouragement. Many participants lived among relatives who surrounded them, and some had migrated from rural areas. Hence, most people in the area were related to one another, which aided building good relationships and a sense of concern for the safety of others. The outcome of these relationships reflected a healthy community atmosphere, particularly in the connections shared with neighbors.

**Category III:** Financial security can be defined as financial status that achieves the necessary balance between daily expenses and income. Participants reported that financial problems caused family happiness to decrease. For example, one participant said:

“The financial security of a family is of primary importance to family happiness. It's very…very important…family happiness depends on the security of the family economy. This is family happiness from my point of view.” (Food vender 2, nuclear family)

Therefore, the participants practiced financial management, including saving money and planning ahead for expenses.

*Subcategory I, saving money*. Most of the participants had low incomes. Hence, they thought carefully about future expenses, buying only essential items, and saving money for emergencies.

“We don't buy useless things…we have to think about what things are essential in our life. We have to know our position…another thing is that we don't want to be in debt in any way.” (Tailor 1, spouse and live apart from their children)

*Subcategory II, planning ahead for expenses*. Participants expressed that they needed to have financial plans to balance their expenses, enabling them to be financially secure.

“(I)…keep thinking about how much money we have to pay for renting the house, daily expenses and… I have money plans for the future…and I have become a disciplined person in terms of money management.” (Security guard, nuclear family)

All the categories described above played an important role, as contextual conditions were influenced by stress. Various coping methods emerged from participants' descriptions, and coping provided an intervention that, if it were adaptive, was able to reduce stress, and vice versa. Therefore, coping methods were defined as participants' ways of releasing tension when they faced difficulties, helping them restore their happiness. Consequently, the family was able to balance life pressures, and function effectively in their daily lives. Participants described various methods for relieving tension.

For example, using a Thai proverb regarding distracting thoughts when faced with marital conflict or family members' arguments, one participant reported the following:

“…Ploiwang (Thai proverb that means “let it be”) … don't think too much…no one can help us; we have to help ourselves first. I've been thinking this way.” (Food vender1, extended family)

Getting monetary support from other relatives to decrease money constraints:

“…(I) am faced with money problems …don't have enough money to pay for rent or expenses… (my siblings) give me money… If they didn't support me, I wouldn't be able to overcome a crisis.” (Security guard, nuclear family)

Participants reported using various coping methods to relieve stress, resulted in a restoration of happiness. Therefore, crises can become opportunities for happiness when coping strategies are employed. Family happiness is shown in Figure 1.

Unhappy families tended to express feelings of unhappiness, as in the following example:

“I don't feel happy at all. My two sons don't talk to each other. They are arguing with each other. I'm not happy about that. I'm trying to talk to them but it's not helping. That makes me shut my mouth. I also take care of him (ex‐husband) and this puts a burden on me (the participant began to cry). Sometimes, my neighbor gives panty for him. This can help a little to save money. My youngest son helps with expenditure, but it's still not enough. I sometimes go out to work and make a small amount of money. I'm not happy at all… (still crying) I live day by day. My family is not happy.”(Housekeeper, nuclear family)

## 5 DISCUSSION

This study demonstrated that family happiness was based on living together without financial problems, maintaining close connections, caring for one another, and financial security. These results are consistent with the findings of a recent study (Lam et al., 2012) in which the family happiness of Chinese participants was found to be composed of the following four components: family harmony, caring and supportive attitudes and behaviors, feeling secure, togetherness, and contentment. When family happiness was challenged by stress over time, participants used coping methods to alleviate the consequences of stress and restore happiness. Previous studies have tended to explore factors related to individual happiness, such as positive emotions, gratitude, and the ability to spread happiness to others (Seligman et al., 2004; Shimai et al., 2006). However, this study shifted the focus from individual happiness to characteristics of family happiness. Thus, the current results can help to elucidate family happiness based on the perspectives of city dwellers.

The findings indicated that close connections played a significant role in family happiness. The participants reported that their happiness was based on family ties, in which they spent time together and talked to one another in a friendly manner. Previous studies reported that family happiness refers to situation in which family members devote sufficient time to each other, love one another, and enjoy family connections (Gray et al., 2013; Stasova & Vilka, 2018). In terms of spending time together, various studies reported that spending time with family members and friends (Helliwell & Wang, 2013; Stasova & Vilka, 2018), in addition to eating together (Yiengprugsawan et al., 2013), affected family happiness. Furthermore, living together and sharing traditional beliefs also represented a means of increasing family happiness (Jones, 2014). One previous study reported that family members sought housing in which family members could interact with one another (Aoki, 2011). Thus, family happiness appears to depend on family connections and talking together, suggesting that family connections and conversations play a significant role in producing a pleasant family atmosphere in which family members experience happiness and strengthen family ties.

This study revealed that caring for one another reflected another characteristic of family happiness, suggesting that participants' actions demonstrating caring and sharing physical/emotional tension enhanced the family atmosphere. Being caring‐oriented enabled family members to gain support and provide encouragement for one another. Consequently, the families of participants in this study were able to overcome difficult situations and maintain family happiness. Previous studies have reported that healthy family relationships are associated with family happiness (Chiang & Lee, 2018; Nanthamongkolchai et al., 2009) and caring for one another (Kramanon & Gray, 2015). In this category, caring for one another also provided ways of building relationships and close connections among neighbors, such as watching out for the safety of others. Similarly, community participation and satisfaction enhanced the happiness of urban dwellers and their communities (Helliwell, Shiplett, & Barrington‐Leigh, 2018; Tangsathapornphanich et al., 2017). Therefore, the findings shed light on how caring can create healthy relationships in terms of relieving the tensions of others in the family and building good relationships with neighbors.

The current findings indicate that financial security is vital to family happiness. The participants reported that they would achieve greater happiness if they were able to manage their expenditure and save money for emergency use. These findings are consistent with previous reports that saving money affects family happiness (Jones, 2014; Lam et al., 2012). One previous study reported that higher income was associated with greater future happiness among Thai families (Sesanu & Singhapakdi, 2014). In addition, several studies found that older people in both Western and Eastern cultures reported that their happiness was based on economic security (Bishop, Martin, MacDonald, & Poon, 2010; Kramanon & Gray, 2015; Royo et al., 2007) and that income status is essential to family happiness (Azizi et al., 2017; Mehrdadi et al., 2016). This study indicates that financial matters are important for family happiness, particularly among people whose incomes are mainly used for daily living expenses such as food, traveling, electricity, and mortgage loans. Hence, participants were focused on saving money and planning for the future, to provide security for future expenses and family happiness.

### 5.1 Limitations

This study explored only one community, in which all participants had low to middle incomes. In addition, none of the participants' family members were experiencing any serious physical or mental illnesses at the time of the study. Hence, caution should be exercised in generalizing the current findings regarding the processes underlying family happiness to other groups in different situations.

## 6 CONCLUSION

The current findings indicate that family happiness is a dynamic process consisting of three categories of features: close connections, caring for one another, and financial security. Family happiness was found to decrease when families were faced with stress. However, families were able to relieve stress using various coping methods. This finding indicates that families take account of familial and social factors that are significant to their happiness, including family atmosphere, connection, and socioeconomic status or conditions.

Potential interventions to build or support families include communications training and coping skills training, both of which are beneficial for enhancing healthy family relationships. In addition, participation in community activities might help families gain close connections with their neighbors, which could, in turn, contribute to supportive and helpful community environments for families. Other possible interventions are related to financial security. Thus, health workers should consider the familial, social, and financial aspects of well‐being. In cases involving unhappy families, health workers should examine families' needs and determine the most effective support for achieving as much happiness as possible.

## AUTHOR CONTRIBUTIONS

Study design: W.N., J.G., S.K.

Data collection: W.N., J.G., S.K., P.P.

Data analysis: W.N., J.G., S.K.

Manuscript writing: W.N., J.G.
